# Steroid-Resistant Immune Thrombocytopenia With Severe Bleeding Successfully Managed Using Eltrombopag and Mycophenolate in a 67-Year-Old Male

**DOI:** 10.7759/cureus.103853

**Published:** 2026-02-18

**Authors:** Abdul Hanan Farooq, Muhammad Badar Khalid, Ayesha Muneer, Labiba Tasfia, Maryam Parvez

**Affiliations:** 1 Acute Medicine, University Hospitals Bristol and Weston NHS Foundation Trust, Weston-super-Mare, GBR; 2 Internal Medicine, University Hospitals Bristol and Weston NHS Foundation Trust, Bristol, GBR; 3 Cardiology, Salford Royal NHS Foundation Trust, Salford, GBR; 4 Medicine, University Hospitals Bristol and Weston NHS Foundation Trust, Bristol, GBR

**Keywords:** eltrombopag, immune thrombocytopenia, mycophenolate mofetil, steroid-refractory itp, thrombopoietin receptor agonist

## Abstract

A 67-year-old man presented with severe mucocutaneous bleeding and profound thrombocytopenia with a platelet count of 6 × 10³ per microliter. Bone marrow examination showed preserved megakaryocytes, and secondary causes were excluded, confirming primary immune thrombocytopenia (ITP). Despite high-dose intravenous methylprednisolone followed by oral corticosteroids, platelet count remained 8 × 10³ per microliter. Two weeks after the presentation, azathioprine was initiated due to persistent severe thrombocytopenia, and eltrombopag was added. Response remained minimal. One month after the presentation, he was classified as having steroid-refractory disease. Azathioprine was discontinued, and mycophenolate mofetil was started. Platelet count rose steadily, reaching 88 × 10³ per microliter at discharge and normalizing to more than 150 × 10³ per microliter within two weeks, with complete resolution of bleeding. This case highlights the diagnostic challenges of ITP in older adults and supports combining thrombopoietin receptor agonists (TPO-RAs) with targeted immunosuppressive therapy in steroid-refractory disease.

## Introduction

Immune thrombocytopenia (ITP) is an acquired autoimmune disorder characterized by isolated thrombocytopenia caused by immune-mediated platelet destruction and impaired platelet production. Its reported annual incidence is approximately three to four cases per 100,000 adults. Bleeding-related morbidity increases with age, and older patients have higher mortality compared to younger cohorts.

ITP in older adults is gaining importance as a clinical concern as the global population continues to age [1]. In individuals over 60, the disease presents additional challenges. These patients often have multiple comorbidities, a higher baseline bleeding risk, and reduced tolerance to treatment-related adverse effects [2].

Corticosteroids remain the standard first-line therapy for newly diagnosed ITP. In older adults, prolonged or repeated steroid exposure is often problematic. These patients have higher rates of metabolic complications, hypertension, osteoporosis, and infections [3]. For patients who fail to respond or who relapse, current guidelines recommend second-line options such as splenectomy, thrombopoietin receptor agonists (TPO-RAs), or immunomodulatory agents. TPO-RAs are often preferred because they provide effective platelet responses with an acceptable safety profile [4]. Real-world data support the long-term effectiveness and tolerability of eltrombopag, a TPO-RA, even in heavily pretreated patients [5].

Despite these advances, evidence remains limited regarding combination or sequential regimens that integrate immunosuppressive agents with TPO-RAs in elderly patients with steroid-refractory ITP. Management becomes more complex when active bleeding or significant comorbidities are present [2,6]. In this report, we describe a 67-year-old male with steroid-refractory ITP who achieved complete and sustained remission using sequential therapy with eltrombopag, a TPO-RA; mycophenolate mofetil, an immunosuppressive antimetabolite; and corticosteroid pulses. This case highlights the importance of clearly defined, age-appropriate therapeutic strategies in refractory ITP.

## Case presentation

A 67-year-old male farmer presented with a two-day history of recurrent nosebleeds occurring two to three times daily, each causing significant blood loss, accompanied by spontaneous bruising over his limbs, abdomen, and oral mucosa. Two months prior, he had been hospitalized for dyspepsia and had taken an unknown course of medications. Prior medications were discontinued. He had no history of liver disease, transfusions, or prior bleeding disorders and was taking folic acid, methylcobalamin, vitamin C, and vitamin E.

On examination, he was alert, oriented, afebrile, and mildly pale. Multiple petechiae were observed on his lower limbs, arms, abdomen, and inner lips. There was no palpable lymphadenopathy, and the abdomen was soft and nontender with no hepatosplenomegaly. Cardiovascular, respiratory, and neurological examinations were unremarkable, and there was no edema or joint swelling.

**Figure 1:**
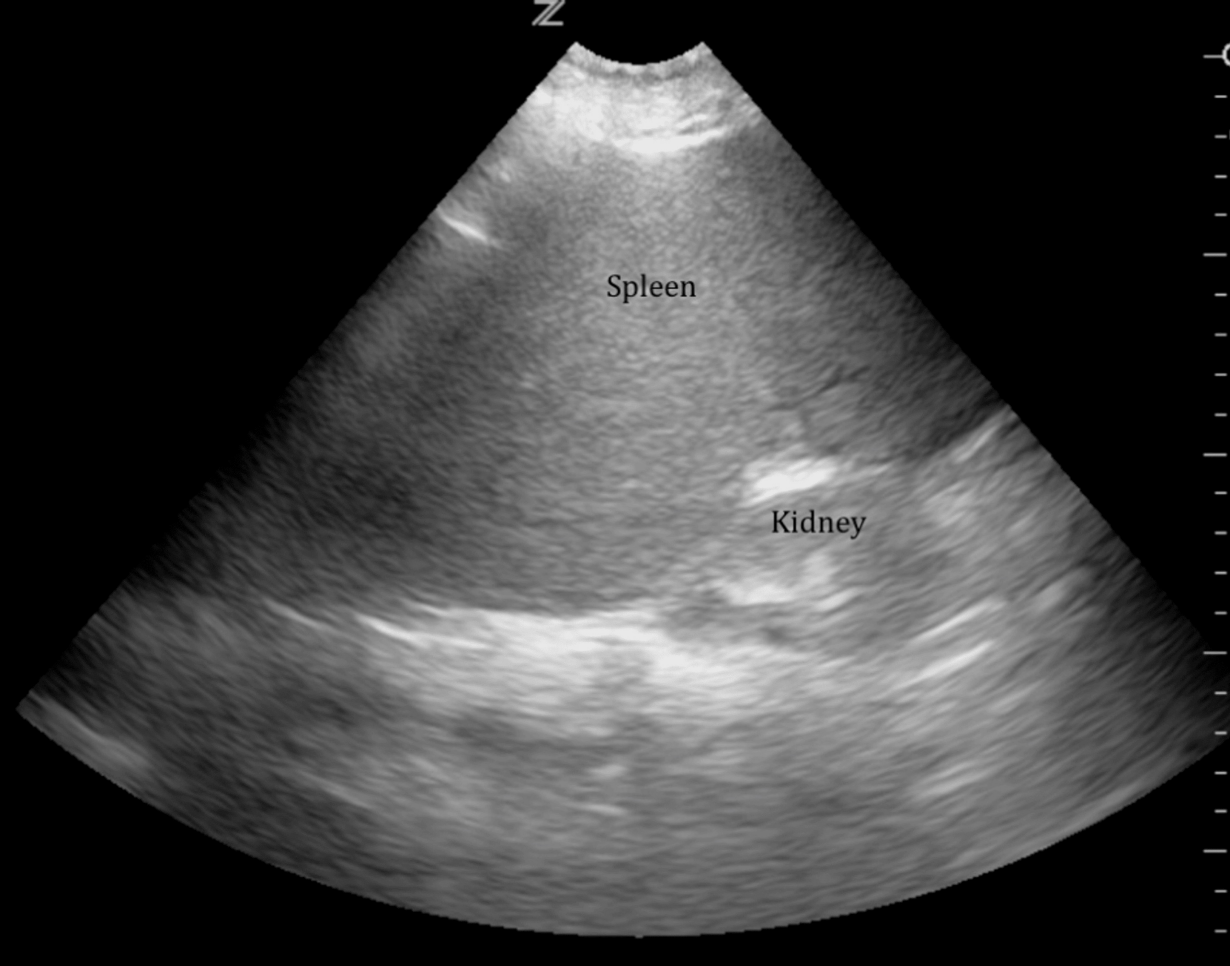
Abdominal ultrasonography Ultrasonography image showing a normally sized spleen with no evidence of splenomegaly or focal lesions.

Laboratory evaluation revealed severe thrombocytopenia with a platelet count of 8 × 10³ per microliter and mild normocytic anemia with a hemoglobin level of 9.7 g per deciliter, with normal leukocyte counts. The reticulocyte count was modestly elevated at 2.1%, while renal and liver function tests were within normal limits. Serology for hepatitis B and C was negative. Abdominal ultrasonography showed no splenomegaly or infiltrative changes (Figure 1). Baseline laboratory parameters are summarized in Table 1.

**Table 1:** Baseline investigations AST: aspartate aminotransferase; ALT: alanine aminotransferase

Parameter	Result	Reference range
Hemoglobin	9.7 g per dL	13 to 17 g per dL
Platelet count	8 ×10³ per µL	150 to 400 ×10³ per µL
Total leukocyte count	Within normal limits	4 to 11 ×10³ per µL
Reticulocyte count	2.1 percent	0.5 to 2.0 percent
Liver function tests	Within normal limits	ALT 7 to 56 U per L, AST 10 to 40 U per L
Renal function tests	Within normal limits	Creatinine 0.6 to 1.2 mg per dL
Hepatitis B surface antigen	Negative	Negative
Anti hepatitis C antibody	Negative	Negative

**Figure 2:**
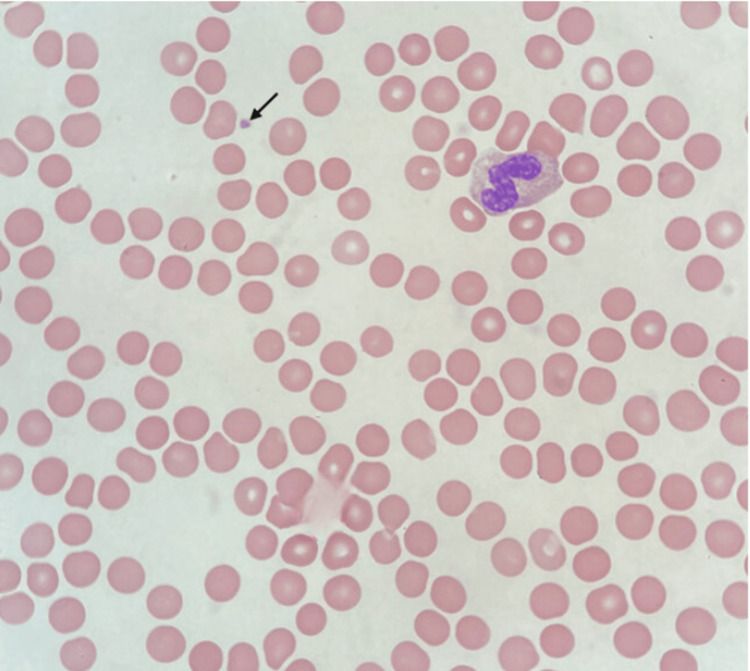
Peripheral blood smear image Peripheral blood smear showing markedly reduced platelets (arrow) with otherwise normal white blood cell morphology.

Peripheral smear demonstrated markedly reduced platelets with anisocytosis, dimorphic red cells, normal white cell morphology, and occasional atypical lymphocytes. These were reactive. There was no progression and no clinical or radiologic evidence of a lymphoproliferative disorder. The findings were suggestive of peripheral platelet destruction. Representative smear findings are shown in Figure 2.

Bone marrow aspirate and trephine biopsy revealed a normocellular marrow with preserved trilineage hematopoiesis, adequate mature megakaryocytes, and reactive lymphoid nodules. Blasts were less than 5%, with no evidence of infiltration or granuloma, confirming a diagnosis of primary ITP.

The patient was initially managed with high-dose intravenous methylprednisolone for five days, followed by oral prednisolone. Despite therapy, platelet counts remained critically low, and he required platelet transfusions for active bleeding, which provided only transient improvement.

Two weeks after the presentation, azathioprine was added as an immunosuppressant, and eltrombopag, a TPO-RA, was initiated. Eltrombopag was started at a dose of 25 mg once daily and escalated to 50 mg after two weeks due to inadequate platelet response. The drug was continued for a total of four weeks. Treatment adherence was confirmed during hospitalization, with no evidence of hepatotoxicity or other adverse effects.

One month after the initial presentation, the patient was classified as having steroid-refractory ITP. Azathioprine was replaced with mycophenolate mofetil. During this period, he developed hospital-acquired pneumonia, which was treated with broad-spectrum antibiotics and later adjusted based on clinical response. Corticosteroid pulses with high-dose dexamethasone were reintroduced for several days. Platelet counts gradually improved over the following days. Serial platelet count trends during treatment are shown in Table 2.

**Table 2:** Platelet count trend over time

Time point	Platelet count (×10³/µL)	Timeline
Day of admission	8	Day 0
After IV methylprednisolone	10	Day 2–3
After eltrombopag initiation	15	Day 5–7
After dexamethasone pulses	50	Day 14
At discharge	88	Day 28
Two-week follow-up	220 (within normal range)	Day 42 (six weeks from initial presentation)

Six weeks from initial presentation, platelet counts rose steadily, reaching 88 × 10³ per microliter at discharge. At the two-week follow-up, platelet counts had normalized completely, and the patient reported no further bleeding episodes. This case highlights the importance of a stepwise approach in managing steroid-refractory ITP in older adults. Sequential therapy with a TPO-RA, targeted immunosuppression, and corticosteroid pulses can lead to sustained remission, even in patients with severe bleeding and an initial poor response to standard therapy.

## Discussion

ITP in older adults presents unique challenges, both diagnostically and therapeutically. Age-related changes in immunity, the presence of comorbidities, and an inherently higher risk of bleeding mean that careful evaluation and individualized management are essential [1,3]. Although older adults with ITP often have multiple comorbidities that can influence treatment choice and tolerability, our patient had relatively few chronic health conditions. This highlights that severe steroid-refractory ITP can occur even in otherwise stable elderly individuals, underscoring the relevance of this case [2].

Corticosteroids remain the first-line therapy for ITP. However, prolonged use in elderly patients can cause significant adverse effects, including high blood pressure, worsening diabetes, osteoporosis, increased infection risk, and neuropsychiatric symptoms [3]. A substantial number of older patients do not respond adequately to corticosteroids, as seen in our patient, which increases the risk of severe bleeding [2,4]. For steroid-refractory cases, current guidelines recommend second-line therapies such as TPO-RAs, immunosuppressive drugs, or splenectomy. TPO-RAs are increasingly preferred in elderly patients because they are both effective and generally well-tolerated [4,7].

Eltrombopag, an oral TPO-RA, has shown high efficacy in elderly patients with chronic or refractory ITP. In real-world studies of patients aged 60 years and older, more than 70% achieved a platelet response, with many attaining complete and sustained remission. Treatment was generally well-tolerated, even in patients with multiple comorbidities or prior therapies [2,5]. While TPO-RAs are effective, some patients do not respond sufficiently to monotherapy. Combining or sequencing TPO-RAs with immunosuppressive agents such as mycophenolate can enhance treatment effectiveness in refractory cases [6,7]. Our patient’s complete and sustained recovery after sequential therapy with eltrombopag, mycophenolate, and corticosteroid pulses illustrates the potential benefits of this approach in elderly patients, particularly those at high risk of bleeding.

This case also highlights the importance of confirming adequate bone marrow function before diagnosing ITP in older adults, as age-related changes or other hematological disorders can complicate the diagnosis [1,3]. Platelet transfusions should be reserved for patients with active bleeding, since they typically provide only temporary benefit and carry potential risks such as volume overload or alloimmunization [1,3]. Overall, our patient’s favorable outcome is consistent with prior evidence demonstrating the efficacy and safety of eltrombopag, both as a single agent and in combination with immunosuppressants, in elderly patients with refractory ITP [2,7]. Further studies are needed to establish standardized protocols and optimize outcomes in elderly patients with steroid-refractory ITP.

## Conclusions

ITP in older adults can be a unique therapeutic challenge. Elderly patients often have reduced tolerance for prolonged corticosteroid therapy and face a higher risk of severe bleeding, making a timely transition to second-line therapies essential. Our patient’s complete remission following sequential treatment with eltrombopag and mycophenolate demonstrates the effectiveness of combining a TPO-RA with an immunosuppressant in cases where steroid therapy alone is insufficient. Although more research is needed to clarify the optimal sequencing and combination of therapies in this population. This case reinforces that a tailored, multi-modal approach can lead to excellent clinical outcomes even in patients with severe, steroid-refractory disease.
